# Dentists' Appointment Book and Pocket Record

**Published:** 1876-01

**Authors:** 


*The Dentists Appointment Book and Pocket Record.*—By E. S. Holmes, D. D. S., of Grand Rapids, Michigan. The following explanation will describe the Record :

"Although the design of "The Dentist's Appointment Book and Pocket Record." must be apparent to nearly every one on examination, yet a few words in explanation of its use may be of value to some. The book contains sixty weeks. Commence the year on the day of the first week on which the year begins by filling the blank—as "Saturday" January 1st, 1876—then the days and weeks will come right for the whole year, leaving out Sundays. The first blank column headed "Time," is for inserting the hours of appointments. The next is for the names, and if necessary addresses of appointees. The next for inserting the kind of operations performed, or to be performed, then the fee for each operation in dollars and cents, and lastly, record on the diagram of the mouth, the exact place where the operation was performed. Where several operations are recorded on one diagram, draw a fine line from the tooth indicated to the margin, and mark on it the hour opposite the patient's name.

When examinations are made previous to appointments, it will be found very convenient to make a record of them, so that when the patient presents himself for operations, a reference to the Record will render another examination unnecessary—thus saving much time. Believing that this little "Appointment Book and Pocket Record," contains more labor-saving conveniences for the dentist, in a more conspicuous and simple form than anything else extant, I offer it to my fellows in dental practice, hoping they will enjoy much pleasure in its use.

